# A novel elicitor MoVcpo is necessary for the virulence of *Magnaporthe oryzae* and triggers rice defense responses

**DOI:** 10.3389/fpls.2022.1018616

**Published:** 2022-10-17

**Authors:** Yanfang Nie, Guanjun Li, Jieling Li, Xiaoshu Zhou, Yanzhi Zhang, Qingchuan Shi, Xiaofan Zhou, Huaping Li, Xiao-Lin Chen, Yunfeng Li

**Affiliations:** ^1^ College of Materials and Energy, South China Agricultural University, Guangzhou, China; ^2^ Guangdong Province Key Laboratory of Microbial Signals and Disease Control, College of Plant Protection, South China Agricultural University, Guangzhou, China; ^3^ State Key Laboratory of Agricultural Microbiology and Provincial Key Laboratory of Plant Pathology of Hubei Province, College of Plant Science and Technology, Huazhong Agricultural University, Wuhan, China

**Keywords:** *Magnaporthe oryzae*, elicitor, vanadium chloroperoxidase, rice, reactive oxygen species

## Abstract

Rice blast caused by *Magnaporthe oryzae* is one of the most important diseases of rice. Elicitors secreted by *M. oryzae* play important roles in the interaction with rice to facilitate fungal infection and disease development. In recent years, several elicitor proteins have been identified in *M. oryzae*, and their functions and importance are increasingly appreciated. In this study, we purified a novel elicitor-activity protein from *M. oryzae*, which was further identified as a vanadium chloroperoxidase (MoVcpo) by MAIDL TOF/TOF MS. The purified MoVcpo induced reactive oxygen species (ROS) accumulation in host cells, up-regulated the expression of multiple defense-related genes, thus significantly enhancing rice resistance against *M. oryzae*. These results suggested that MoVcpo functions as a pathogen-associated molecular pattern (PAMP) to trigger rice immunity. Furthermore, *MoVcpo* was highly expressed in the early stage of *M. oryzae* infection. Deletion of *MoVcpo* affected spore formation, conidia germination, cell wall integrity, and sensitivity to osmotic stress, but not fungal growth. Interestingly, compared with the wild-type, inoculation with *MoVcpo* deletion mutant on rice led to markedly induced ROS accumulation, increased expression of defense-related genes, but also lower disease severity, suggesting that MoVcpo acts as both an elicitor activating plant immune responses and a virulence factor facilitating fungal infection. These findings reveal a novel role for vanadium chloroperoxidase in fungal pathogenesis and deepen our understanding of *M. oryzae*-rice interactions.

## Introduction

Rice is the most important food staple for more than half of the world’s population (Salman et al., 2022). Rice blast, caused by *Magnaporthe oryzae*, is one of the most destructive rice diseases worldwide, which results in substantial crop loss and potential economic disaster. Breeding of blast-resistant cultivars and chemical control are the major strategies to control the disease. However, it is difficult to obtain stable resistant cultivars because of the high pathogenic diversity and rapid emergence of new races. Fungicide application usually results in environmental pollution and fungicide resistance in *M. oryzae*. The increasing number of studies on the molecular pathogenicity of *M. oryzae* has contributed to better understanding of the disease and may offer important insights into the development of more efficient and safe control strategies.

In response to pathogen attacks, plants have evolved at least two different levels of active defense systems to protect themselves. Extracellular recognition of conserved pathogen-associated molecular patterns (PAMPs) leads to the first layer of inducible defenses, termed pattern-triggered immunity (PTI) (Boutrot and Zipfel, 2017). On the other hand, to overcome plant immunity, pathogens deliver effectors into plant cells to suppress PAMP-triggered immunity (Jones and Dangl, 2006). Many effectors can also be recognized by plant resistance proteins, which is referred to as effector-triggered immunity (ETI) (Park et al., 2012). The co-evolution between plants and pathogens has been described as a zigzag model by Jones and Dangl (2006). Recent studies show that some PAMPs and effectors are described as elicitors, which induce rapid and enhanced defense responses in plants, promote the production of new antibiotics and bioactive metabolites, and lead to plant resistance against pathogen infection (Alhoraibi et al., 2019; Héloir et al., 2019; Zong et al., 2021). Therefore, while the term “elicitor” was originally used for molecules capable of inducing the production of phytoalexins, it now generally refers to compounds stimulating any type of plant defense (Thakur and Sohal, 2013; Abdul Malik et al., 2020; Ochoa-Meza et al., 2021).

Numerous types of elicitors, including proteins, glycoproteins, sphingolipid, chitin, *N*-acetylchitooligosaccharide, and oligosaccharides, have been identified from mycelial cell walls, conidia and culture filtrate of *M. oryzae* (Qiu et al., 2009; Nie et al., 2019). Two sphingolipid elicitors (cerebrosides A and C) have been shown to trigger phytoalexin accumulation and hypersensitive cell death in rice plants (Koga et al., 1998). Two glycoproteins have been demonstrated to induce resistance responses in rice (Kanoh et al., 1993; Schaffrath et al., 1995). PemG1 has been reported to activate SA- and Ca^2+^-related signaling pathways and induce systemic acquired resistance in plants (Peng et al., 2011). Recently, two protein elicitors (MoHrip1 and MoHrip2) are well-characterized to activate early defense signaling in tobacco and enhances rice resistance to *M. oryzae* (Chen et al., 2012; Chen et al., 2014; Nie et al., 2019). However, only a limited number of elicitors have been characterized in *M. oryzae* so far and much remains unclear in elicitor functions because most elicitors lack a conserved motif or domain.

In this work, we report the purification, identification, and characterization of a novel protein elicitor named as MoVcpo from *M. oryzae* using a combination of ultrafiltration, chromatography, and MAIDL-TOF/TOF spectrometry. Exogenous treatment of rice with MoVcpo resulted in induced plant defense in tobacco plants and enhanced rice resistance against *M. oryzae* infection. The homologous recombination strategy was used to knock out *MoVcpo* for functional investigation. The *MoVcpo* deletion mutant exhibited reduced virulence on rice plants and activated multiple plant defense responses. This study will contribute to a better understanding of the molecular basis of elicitor-induced plant defense against fungal pathogens.

## Materials and methods

### Fungus and plant

*Magnaporthe oryzae* race ZC13 was used as the wild-type control in this study, which is one of the primary blast races in Guangdong Province. The fungi cultured on YDA medium for 10 d and was subsequently cultured in YPD liquid medium for 14 days in the dark at 25°C to maintain the mycelium. The fungal conidia were prepared according to the methods described previously (Li et al., 2015). The conidia were adjusted to 5×10^4^ conidia/mL for further use. Two rice cultivar (*M. oryzae*-susceptible CO39 and -resistant C101LAC) and tobacco (*Nicotiana benthamiana*) were used in this study (Li et al., 2014). Plants were grown in a greenhouse at 25 ± 1°C, 70-80% relative humidity with a 12-h light/dark photoperiod as described (Li et al., 2012). Rice seedlings at the fourth-leaf stage and five-week tobacco plants were used for further experiments.

### Purification of the elicitor

The mycelia were harvested on Whatman filter paper (0.45 μm, Millipore, Billerica, MA, USA) and washed with deionized H_2_O four times on a Buchner funnel, weighed and frozen at -20°C. The crude elicitor fraction (CEF) was prepared according to the methods as described previously (Kanoh et al., 1993; Griffin, 1996). CEF was desalted and concentrated through ultrafiltration using a 50-mL Amicon Ultra Centrifugal Filter Device with a molecular weight cutoff of 10 kDa (Millipore, Billerica, MA, USA), and lyophilized for further use. The lyophilized CEF was redissolved in 2 mL of 0.02 mol/L Tris-HCl buffer (pH 7.2) (TH buffer). After centrifugation at 10,000×g for 10 min, the supernatant was applied to a DEAE-Sepharose FF anion exchange column (1.9 cm ×27 cm, Pharmacia, Sweden), which was pre-equilibrated with TH buffer. Unbound material was removed using TH buffer, and adsorbed fractions were eluted with a linear NaCl gradient (0.05-0.75 mol/L in TH buffer) at a flow rate of 0.35 mL/min. All fractions were collected according to the elution profile and dialysed against TH buffer overnight prior to the determination of elicitor activity. The fractions showing the maximum elicitor activity was further loaded onto an Sephadex G-100 column (1.9×58 cm, Pharmacia, Sweden), which was eluted with the same TH buffer. Elicitor activity fractions were collected and applied to a ConA-Sepharose 4B column (1.9×27cm, Pharmacia, Sweden), which was pre-equilibrated with 0.02 mol/L TH buffer containing 0.2 mol/L NaCl, 1 mmol/L MgCl_2_, 1 mmol/L CaCl_2_ and 1 mmol/L MnCl_2_. The bound fractions were eluted with the equilibration buffer containing 0.05 mol/L α-D-glucose and a linear gradient of 0.05-0.15 mol/L α-D-mannose. The elicitor-activity fraction was collected and dialysed against TH buffer overnight, lyophilized, and resuspended in 0.02 mol/L TH buffer. Protein concentration was determined using the Bradford method, with BSA as the standard (Bradford, 1976). All purification steps were carried out at 4°C. The ability of the elicitor to induce the phenylalanine ammonia-lyase (PAL) and peroxidase (POD) activity in rice was used as an indicator during the entire purification process as described previously (Schaffrath et al., 1995).

### Characterization of the elicitor

The purity and molecular weight (MW) of the elicitor was determined by SDS-PAGE, which was stained with the Coomassie brilliant blue G-250 (CBB) staining method. To detect whether the elicitor contains carbohydrate moieties, the gels were also visualized by the periodic acid-Schiff staining (PAS) as previously described (Van-Seuningen and Davril, 1992). Thermal stability was determined by incubating the elicitor at 4, 20, 30, 50, 60, 80, and 100°C for 30 min, then tested for elicitor activity. Stability at low and high pH was tested by incubating the elicitor in solutions of pH 2, 5, 6, 7, 8 and pH 12 for 12 h at 25°C, respectively. The samples were then dialyzed against TH buffer for 12 h to remove excess HCl and NaOH. Enzymatic digestion (0.1 mg/mL trypsin) (Promega, Madison, WI, USA) of the elicitor was carried out in TH buffer for 30 min at 37°C as previously described (Schaffrath et al., 1995). Periodate oxidation of the elicitor was carried out according to the procedure of De Wit and Roseboom (1980) by incubation in 20 mmol/L NaIO_4_ for 24 h at 4°C in the dark, and excessive NaIO_4_ was destroyed by adding ethylene glycol to the samples. To determine the effect of the elicitor on spore germination and mycelial growth of *M. oryzae in vitro*, filter-sterilized elicitor solution was added to YDA medium. conidial suspensions of *M. oryzae* were then spread to the plates and incubated in the dark at 28°C. Spore germination was observed at 3 and 6 h after inoculation, respectively. The colony diameter was determined 7 days after inoculation (dpi) on the YDA medium with a 3-mm-diameter plug of *M. oryzae* mycelium. An equal volume of TH buffer was added to YDA medium, serving as a control.

### Identification of the elicitor by MALDI-TOF/TOF

The elicitor protein band was excised from the CBB-staining SDS-PAGE gel, and in-gel digestion of proteins was performed as previously (Li et al., 2015). MS and MS/MS spectra were conducted using the 5800 MALDI-TOF/TOF™ Analyzer (AB Sciex, Framingham, Massachusetts, USA). The MS together with MS/MS spectra were searched using MASCOT engine (Matrix Science, London, UK; version 2.4) against the NCBI_*Magnaporthe oryzae* database.

### Gene disruption and complementation

The knockout vector for *MoVcpo* was constructed according to the standard principle of homologous recombination. Briefly, the upstream and downstream flanking sequences of *MoVcpo* were generated by PCR using chromosomal DNA of *M. oryzae* race ZC13 as template. The upstream and downstream sequence was respectively digested by *Kpn*I/*Apa*I and *Eco*RI/*Xba*I, then respectively inserted into the of *Kpn*I-*Xba*I site of pCT74 vector to form the knockout construct pCT74-*MoVcpo*-KO. PEG-mediated protoplast transformation was conducted in this study. The deletion mutants were generated by transforming the construct pCT74-*MoVcpo*-KO into the wide type and screened by resistance to hygromycin B, PCR, southern blot, and RT-qPCR analysis. To further verify the function of *MoVcpo*, gene complementation was conducted by protoplast transformation of *MoVcpo*-deleted mutant with the complementation vector pCTZN (Zeocin resistance), which was constructed by introducing the entire coding region of *MoVcpo* with its native promoter and terminator. The putative complementation mutants were selected with zeocin, further confirmed by PCR. Primers used in plasmid construction and qPCR are listed in **Supplementary Table 1**.

### Stress sensitivity assays

To test fungal response to environmental stresses, strains were cultured on YDA medium supplemented with the final concentrations of 0.8 mol/L NaCl, 0.8 mol/L sorbitol, 0.01% w/v SDS, 200 μg/mL congo red (CR), 200 μg/mL calcofluor white (CFW) or 20 mol/L H_2_O_2_ for 5 d at 28°C. The experiments were repeated three times.

### Inoculation of the elicitor of *N. benthamiana*

Five μL of the elicitor solution (50 μg/mL) was infiltrated into *N. benthamiana* leaves. BAX and TCTP were amplified by PCR and inserted into the pBI121 vector, respectively. The pBI121 vectors containing BAX and TCTP were transformed into *Agrobacterium tumefaciens* strain GV3101 through electroporation method and then transiently expressed in *N. benthamiana* leaves according to the methods of Ma et al. (2012), which were used as positive and negative controls, respectively. Each treatment was performed on three leaves from six individual plants, and the assay was repeated at least three times. The inoculated leaves were photographed in 3-4 d after infiltration.

### RT-qPCR analysis

Total RNA from *M. oryzae* was extracted using a Fungal RNA kit (Omega, USA) according to the manufacturer’s protocol. Total RNA from tobacco and rice leaves was extracted using Plant RNA Kit (Omega, USA) following the manufacturer’s instructions. The RT-qPCR was performed on a CFX Coxnnect™ Real-Time System (Bio-Rad, Hercules, CA, USA) with the SYBR Premix Ex Taq Kit (TaKaRa, Beijing, China) according to the manufacturer’s instructions. For *M. oryzae*, the *MoPot2* gene was used as an internal control. For tobacco and rice, *NbEF1a* and *OsUbiquitin* were used as references, respectively. All gene-specific primers used are listed in **Supplementary Table 1**. The relative changes in gene expression levels were calculated using the 2^-ΔΔC^ *^t^* method (Rajeevan et al., 2001). Each sample was represented by three independent biological replicates.

### Inoculation of the elicitor and *MoVcpo* deletion mutants of rice seedling

For enzyme activity assay, 5 μL of the elicitor solution (50 μg/mL) was mounted onto a punch-inoculated spot of 2 mm diameter on the unexcised 4^th^ leaf. The activity of LOX (lipoxygenase), APX (ascorbate peroxidase), SOD (superoxide dismutase), and CAT (catalase), was determined according to the methods described previously (Li et al., 2014). For ROS determination, elicitor solution was sprayed onto rice leaves. For disease severity assay, the elicitor-pretreated rice seedlings were further spray-inoculated with fresh *M. oryzae* spores at the 2^nd^ day. ROS was determined as described previously (Li et al., 2015). For inoculation of *MoVcpo* deletion mutants, spores were applied by spraying to determine disease severity (Li et al., 2014), or by punch inoculation (Park et al., 2012) to determine the blast sporulation rate on the lesions and the fungal biomass. Disease symptoms were recorded 7 d after inoculation and disease index were calculated by the formula: Disease Index = [Σ(no. of plants of each grade × disease grade)/(total no. of plants × the highest disease grade)] ×100 (Li et al., 2012). Relative fungal biomass was performed by DNA-based qPCR and calculated as a ratio (*MoPot2*/*OsUbiquitin*) represented by the equation 2^(CT(^ *^OsUbiquitin^* ^)-CT(^ *^MoPot2^* ^)]^ as previously described (Park et al., 2012). All the experiment was repeated three times.

### Statistical analysis

Statistical analysis was carried out using the SPSS software (Release v14.0; SPSS Company, USA). Analysis of covariance (ANCOVA) followed by Duncan’s multiple range test was used to carried out quantitative analysis of the *M. oryzae*-regulated proteins, H_2_O_2_ production, enzyme activities and qRT-PCR. The data were presented as mean ± SE (standard error).

## Results

### Purification, identification and characterization of a putative elicitor MoVcpo

**Figure 1:**
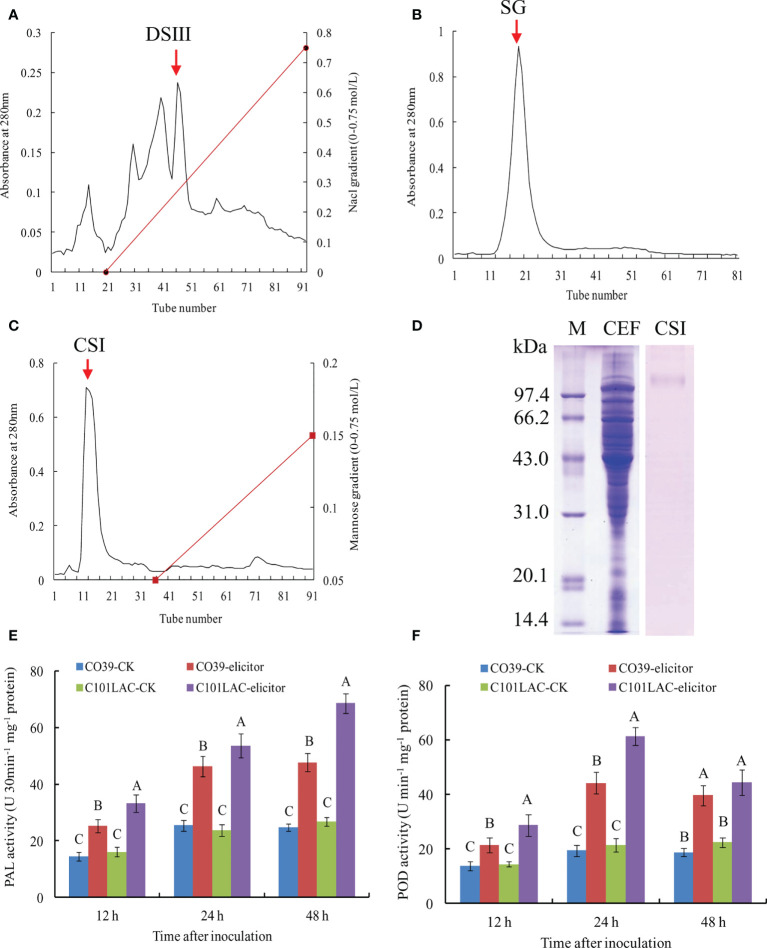
Purification of MoVcpo from *M. oryzae*. **(A)** Chromatography on DEAE-Sepharose FF of the crude elicitor fraction. Elution was performed with a NaCl gradient. **(B)** Gel permeation chromatography on Sephedex G-75 of DEAE-Sepharose FF fraction DSIII. **(C)** Chromatography on ConA-Sepharose 4B of Sephadex G-75 fraction SG. Elution was performed with a mannose gradient. DSIII, SG and CSI were indicated by an arrow. Absorbance of the material was monitored at 280 nm. **(D)** SDS-PAGE analysis of the purified elicitor protein using CBB staining. Fifteen μg total proteins per lane was loaded. Lane M, marker; lane CEF, the crude elicitor fraction; lane CSI, the purified elicitor. **(E)** PAL activity of rice leaves induced by the purified elicitor. **(F)** POD activity of rice leaves induced by the purified elicitor. Values are the means ( ± SE) based on three independent experiments and bars indicate standard deviations. Different letters indicate statistical significance (*p* < 0.05) using Duncan’s new multiple range method.

In order to identify proteins with elicitor activity, the vegetative mycelium of *M. oryzae* was incubated in YPD liquid medium. The mycelium was harvested and broken up mechanically. After centrifugation, celite535 filtration, and PM-10 membrane ultrafiltration, a CEF fraction (>10 kDa) was obtained, which exhibited high elicitor activity (Data not shown). The CEF fraction was further purified with DEAE-Sepharose FF chromatography, and the elution profiles were shown in **Figure 1A**. We evaluated the elicitor activity of various peak fractions, among which DSIII (indicated by an arrow) showed the highest elicitor activity. After Sephadex G-75 chromatography of DSIII, the main peak fraction (SG) was obtained (**Figure 1B**). SG was passed over a ConA-Sepharose 4B column and the main peak fraction CSI was obtained, which was not bound to the column (**Figure 1C**). The CSI fraction displayed a single band in SDS-PAGE analysis with CBB staining, with a relative molecular weight of 101 kDa (**Figure 1D**). CSI fraction also exhibited only a band in SDS-PAGE analysis with PAS staining for carbohydrate moiety (**Supplementary Figure 1**), indicating that CSI may be a glycoprotein. When induced with the CSI fraction, significant increases in PAL and POD activities were observed in both the susceptible rice cultivar CO39 and the resistant cultivar rice C101LAC during the early stage of inoculation (12~48 h) (**Figures 1E, F**; **Supplementary Figure 2**). Because PAL and POD have been widely used as biomarkers of rice defense against pathogens (Schaffrath et al., 1995; Wang et al., 2019), we concluded that the CSI fraction can function as an elicitor.

The purified CSI fraction was further applied to preparative SDS-PAGE, and stained with CBB staining. The single protein band was excised for tryptic digestion and further analyzed by MALDI-TOF/TOF MS. The spectra of the protein are provided in **Supplementary Figure 3**. Finally, the protein was identified as a vanadium chloroperoxidase (named MoVcpo) with high confidence. We then conducted bioinformatic analyses to characterize the sequence features of MoVcpo (GenBank accession No. ELQ63332.1). The gene encodes a 595 amino acid protein with two protein domains, a VCPO_N domain and an acidPPc domain (**Supplementary Figure 4A**). SignalP, 5 DeepTMHMM, and NetGPI did not detect any signal peptide, transmembrane domain, or glycosylphosphatidylinositol (GPI) anchor signal in MoVcpo. Subcellular localization prediction by ProtComp further suggested that MoVcpo is an extracellular secreted protein. A BlastP search against the NCBI non-redundant protein database revealed that MoVcpo shared high levels of sequence similarity with several proteins of plant fungal pathogens, including hypothetical protein PspLS_07003 (TLD22960.1; similarity: 92.74%) from *Pyricularia* sp. CBS 133598, vanadium chloroperoxidase (XP_009227161.1; similarity: 78.99%) from *Gaeumannomyces tritici* R3-111a-1, hypothetical protein S7711_06685 (KEY74787.1; similarity: 71.19%) from *Stachybotrys chartarum* IBT7711. Phylogenetic analysis showed that MoVcpo shared above 75% sequence identifies with several proteins of fungal pathogens, including *Pyricularia* sp., *G. tritici*, and *P. pennisetigena* (**Supplementary Figure 4B**).

MoVcpo was stable at 4, 25 and 30 and 50°C for 30 min and retained its biological activity of inducing PAL activity. However, MoVcpo was thermally denatured with a loss of biological activity at 60, 70, or 80°C for 30 min (**Supplementary Figure 5A**). MoVcpo also retained the elicitor activity at pH 5, 6, 7 or 8 for 12 h, but lost the activity at pH 2 and 12 for 12 h (**Supplementary Figure 5B**). Enzymatic and chemical digestions were further conducted to evaluate the nature of the elicitor-active component. The activity of MoVcpo was not affected by digestion with NaIO_4_, indicating that the carbohydrate moiety is not necessary for elicitor activity. When the protein moiety was destroyed with trypsin, the elicitor activity was completely abolished (**Supplementary Figure 5C**). These results suggest that the protein moiety is necessary for elicitor activity.

### MoVcpo activates plant immune responses in *N. benthamiana*

**Figure 2:**
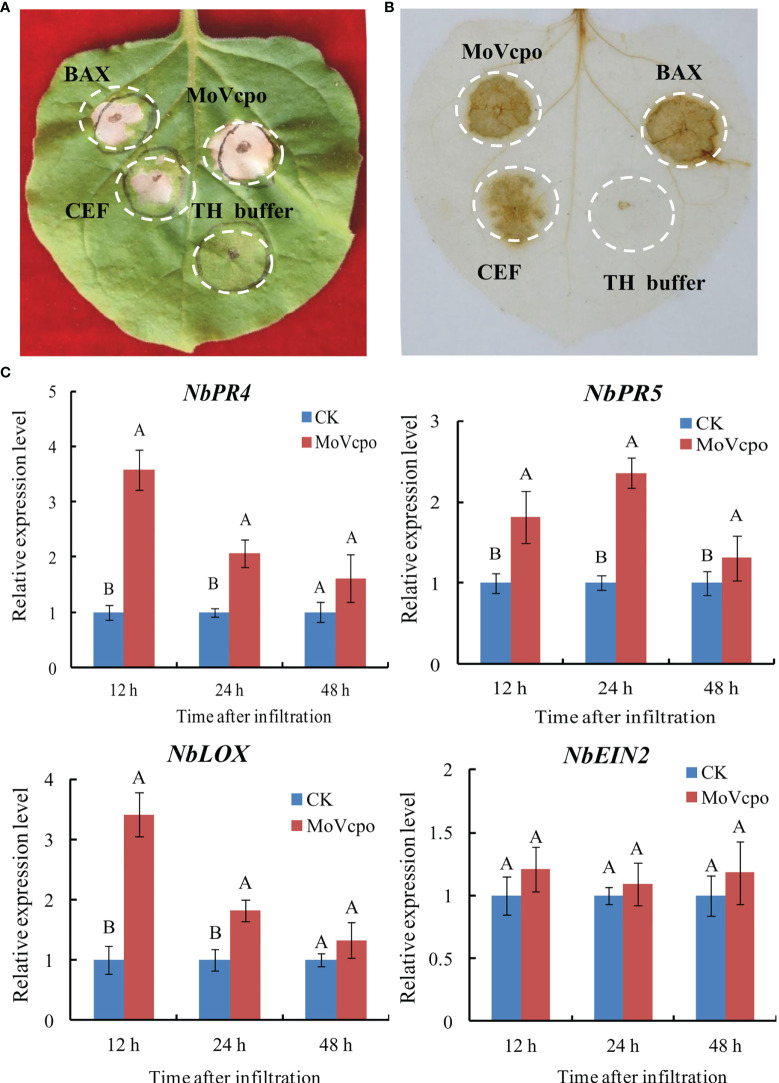
MoVcpo induces plant immune responses in *Nicotiana benthamiana.* **(A)** MoVcpo induced the hypersensitive reaction in *N. benthamiana* leaves. Tobacco leaves were infiltrated with CEF (crude elicitor fraction, 50 μg/mL), MoVcpo (50 μg/mL), *A*. *tumefaciens* expressing BAX (as positive control) or TH buffer (as negative control). The leaves were photographed 3-4 d after infiltration. **(B)** Reactive oxygen species were detected by DAB staining. **(C)** RT-qPCR analysis of four defense-related genes after infiltration with MoVcpo solution (50 μg/mL). Values are the means ( ± SE) based on three independent experiments and bars indicate standard deviations. Letters indicate statistical significance (*p* < 0.05) using Duncan’s new multiple range method.

To evaluate the elicitor activity of MoVcpo, CEF and MoVcpo solutions were respectively infiltrated into tobacco leaves. The results showed that both CEF and MoVcpo treatments resulted in strong hypersensitive reaction (HR) activity in *N. benthamiana*, and the latter induced larger necrotic area of the tissue (**Figure 2A**). Consistent with the HR reaction, DAB staining showed that both CEF and MoVcpo could induced ROS production in *N. benthamiana* leaves, with BAX as a positive control and TH buffer as a negative control (**Figure 2B**). The expression of four defense-related marker genes, namely *NbPR5*, *NbPR4*, *NbLOX*, and *NbEIN2*, were further examined *via* RT-qPCR after MoVcpo infiltration (**Figure 2C**). The expression levels of the SA-dependent defense genes (*NbPR5* and *NbPR4*) and JA-dependent defense gene (*NbLOX*) were increased significantly after MoVcpo treatment (**Figure 2**). In contrast, the expression of *NbEIN2* for ethylene-dependent defense remained stable after treatment. Taken together, the results of ROS production and defense gene expression analyses suggest that MoVcpo may induce plant immune responses by activating the SA- and JA- mediated defense pathways.

### MoVcpo enhances rice resistance against *M. oryzae* infection

**Figure 3:**
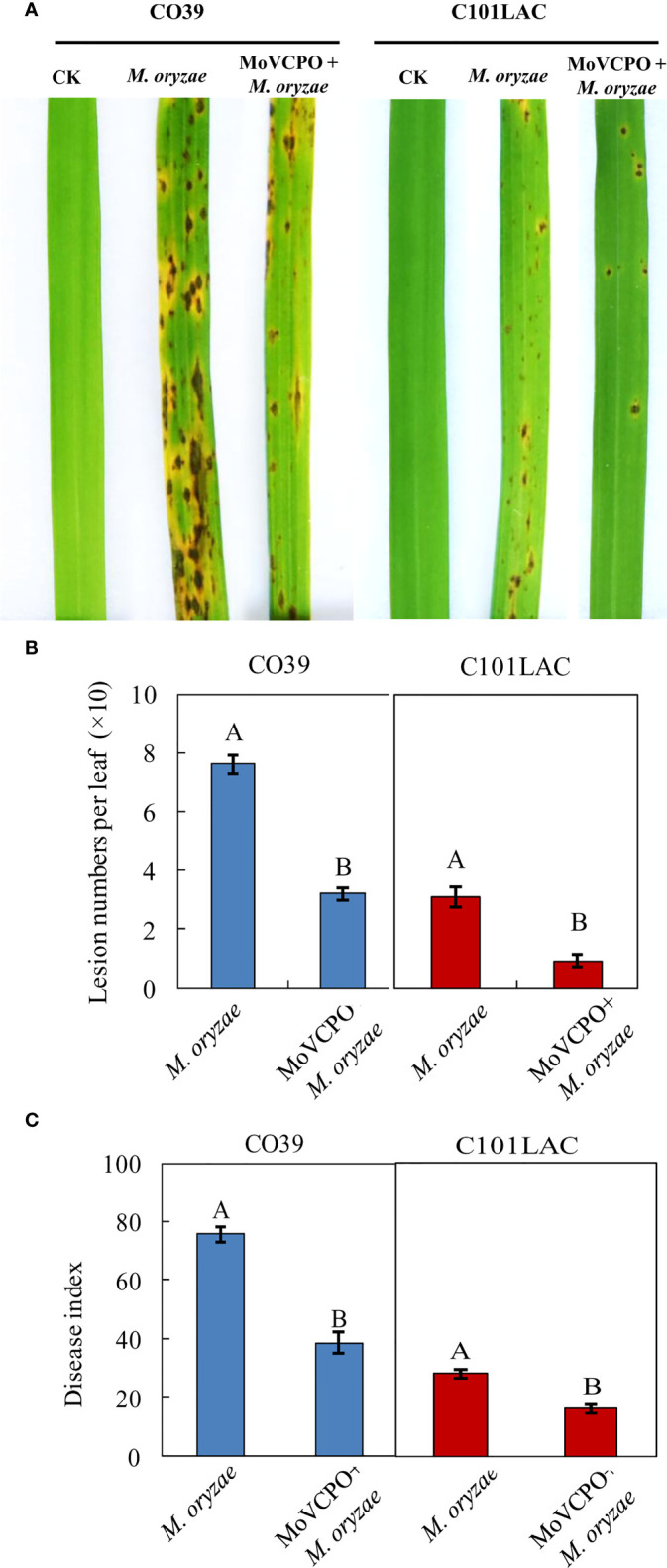
MoVcpo enhances rice resistance against *M. oryzae* infection. **(A)** Disease symptoms. Rice seedlings were sprayed with 50 μg/mL MoVcpo solution or TH buffer as a control at 2 d before inoculation with *M. oryzae*. Disease severity was scored at 7 d post inoculation with conidia and the leaves of representative plants were photographed. **(B)** Disease index. **(C)** Mean number of lesions per leaf. Data bars are the means (± standard error) of 90 replicates from three independently biological experiments. The letters above the bars are significantly different at 0.05 level. All experiments were repeated for three times.

To determine the optimal concentration of MoVcpo for subsequent investigation of induced rice resistance to *M. oryzae*, rice plants were inoculated with *M. oryzae* 48 h after treatment with various concentrations of MoVcpo. Quantitative analysis showed that, when treated with concentrations of MoVcpo as low as 10 μg/mL, there was a significant reduction in disease index in MoVcpo-treated plants compared with the water-treated control (**Supplementary Table 2**). When the concentrations of MoVcpo was ≥50 μg/mL, the disease reduction was no longer significantly increased in both of C101LAC and CO39 lines. Thus, 50 μg/mL was selected as the optimal concentration of MoVcpo for induction of disease resistance and employed in subsequent experiments. When pretreated with 50 μg/mL MoVcpo, a significant reduction in disease symptoms was observed in both C101LAC and CO39 (**Figure 3A**), with the lesion number on rice leaves caused by *M. oryzae* reduced by about 2.4- and 3.3-fold (**Figure 3B**), and the disease index decreased by 49.0% and 42.1% in CO39 and C101LAC, respectively (**Figure 3C**). To test whether the protective role of MoVcpo on rice was due to its effect on *M. oryzae* growth, we performed an inhibitory test on fungal growth and observed little inhibitory effect on hyphal growth even at the highest concentration (100 μg/mL) of MoVcpo (**Supplementary Figure 6A**). Likewise, spore germination was also not affected by MoVcpo treatment up to 100 μg/mL treatment (**Supplementary Figure 6B**). Taken together, the above findings indicate that the protective role of MoVcpo on rice was due to its activity in the induction of rice resistance to *M. oryzae*.

### MoVcpo activates ROS accumulation in rice leaves

**Figure 4:**
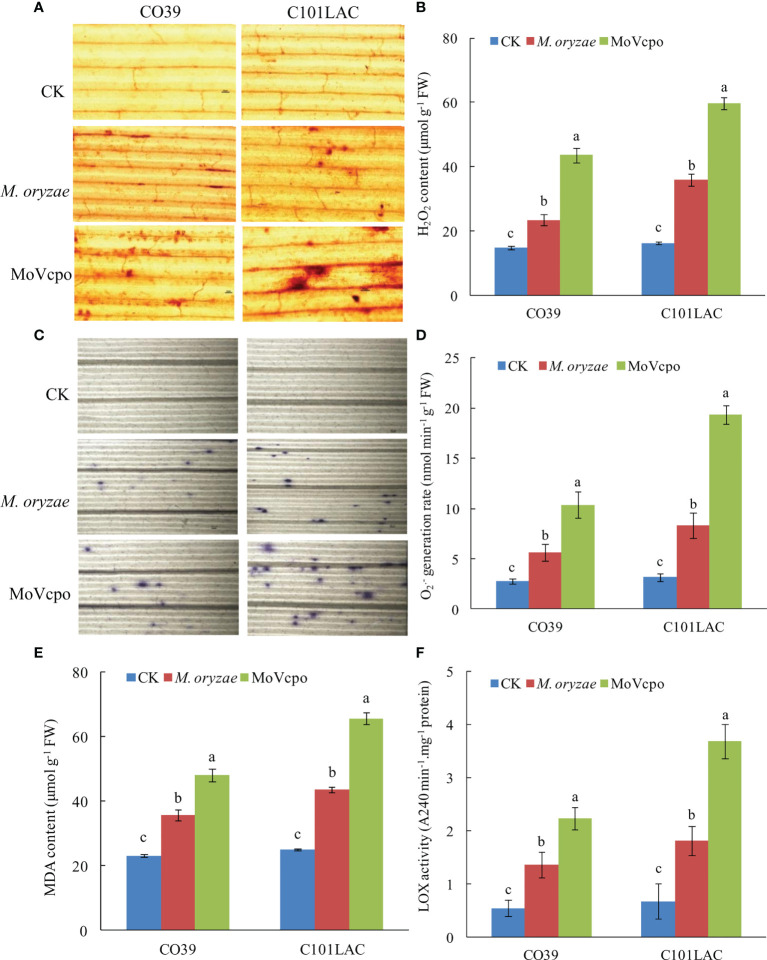
ROS accumulation in rice leaves induced by MoVcpo. **(A)** H_2_O_2_ production detected by DAB staining at 24 h after inoculation. **(B)** Quantitative analysis of H_2_O_2_ content in rice leaves. **(C)** O_2_^.-^ production detected by NBT staining at 24 h after inoculation. **(D)** Quantitative analysis of O_2_^.-^ content in rice leaves. **(E)** MDA contents. **(F)** LOX activity. Arrowheads indicate the stained spots in rice leaves. Experiments were repeated at least three times. Values are the means from three independent experiments and bars indicate standard deviations. The letters above the bars are significantly different at 0.01 level.

ROS production is critical for responding to biotic and abiotic stresses (Castro et al., 2021). Our qualitative and quantitative analyses showed that ROS accumulation in the rice leaves was dramatically induced by MoVcpo (**Figure 4**). Detection of H_2_O_2_ production by using DAB staining showed significant accumulations of H_2_O_2_ in the vascular tissues in both rice cultivars at 24 h after treatment with 50 μg/mL MoVcpo (**Figure 4A**). Consistent with the histochemical staining results, MoVcpo treatment resulted in an increase in H_2_O_2_ content by 2.69 and 3.49 times in CO39 and C101LAC, respectively (**Figure 4B**). Similarly, NBT staining assays revealed increased O_2_^.-^ production in both cultivars after MoVcpo treatment (**Figure 4C**). Relative to the corresponding water-treated controls, MoVcpo treatment resulted in 4.21- and 4.98- times O_2_^.-^ content in CO39 and C101LAC, respectively (**Figure 4D**). Malondialdehyde (MDA) has been widely used as a convenient biomarker of oxidative stress, specifically for lipid peroxidation (Ayala et al., 2014). Significant increases in MDA contents were also detected in both CO39 and C101LAC upon MoVcpo treatment (**Figure 4E**). Lipoxygenase (LOX) reaction is a possible source of ROS and other radicals. We found that the LOX activity was increased to 5.5-fold in C101LAC and 4.1-fold in CO39 after MoVcpo treatment (**Figure 4F**), suggesting that MoVcpo treatment led to higher activity of LOX in C101LAC than in CO39. The results also showed that the contents of H_2_O_2_, O_2_^.-^ and MDA in rice leaves were lower than those in MoVcpo inoculated samples, but higher than those in control samples upon *M. oryzae* inoculation.

### MoVcpo induced defense-related genes expression in rice seedlings

**Figure 5:**
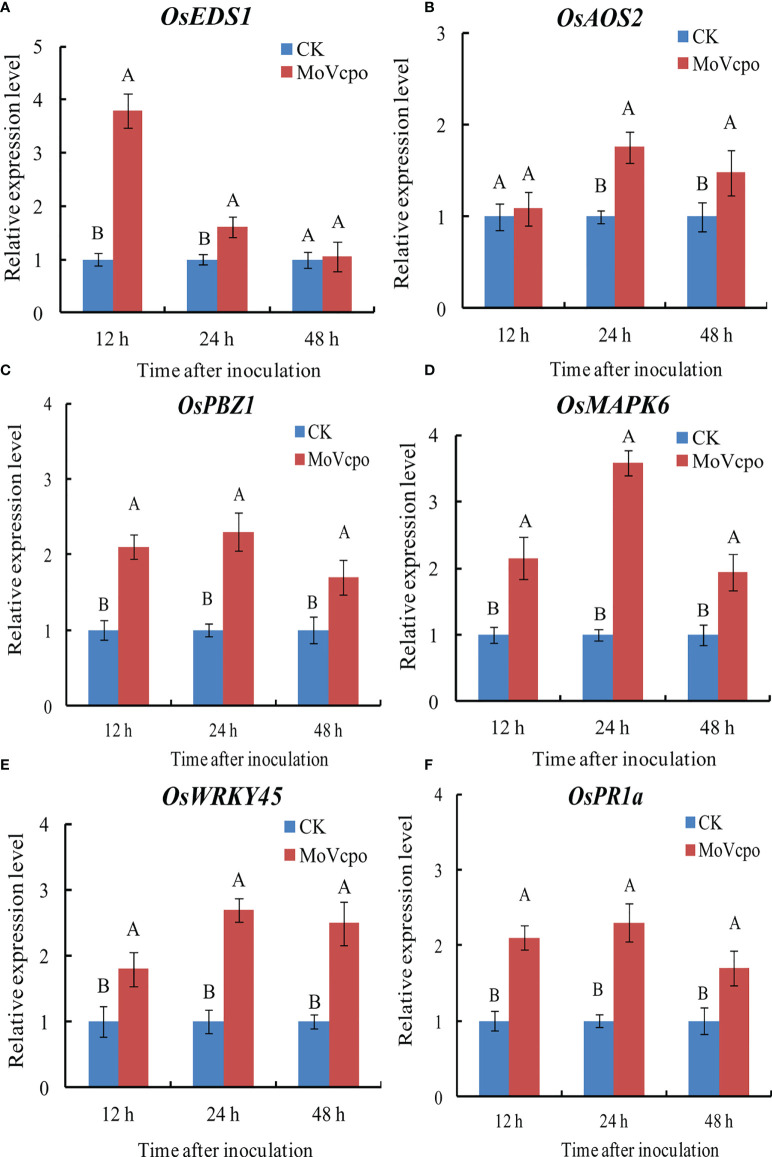
Transcription patterns of *OsEDS1* **(A)**, *OsAOS2* **(B)**, *OsPBZ1* **(C)**, *OsMAPK6* **(D)**, *OsWRKY45* **(E)**, and *OsPR1a* **(F)** in MoVcpo-treated leaves as determined by RT-qPCR. The rice constitutive gene *Osactin* was used as internal reference. Values are the means ( ± SE) based on three independent experiments and bars indicate standard deviations. Different letters indicate statistical significance (*p* < 0.05) using Duncan’s new multiple range method.

To elucidate the molecular mechanisms underlying the general defense response of rice seedlings upon MoVcpo treatment, we evaluated the expression of the marker genes for SA-signaling pathway (*OsEDS1*, *OsWRKY45*), JA-signaling pathway (*OsAOS2*, *OsPBZ1*), MAPK signaling pathway (*OsMAPK6*), and selected genes encoding PR protein (*OsPR1a*). We compared the expression of these genes in rice seedlings inoculated with MoVcpo or TH buffer, and found that almost all of them exhibited significantly increased expression at all three time points after the MoVcpo treatment. In addition, except for *OsEDS1*, all other genes exhibited their greatest levels of up-regulation at 24 h after inoculation (**Figure 5**). Consistent with the induced plant immunity response in *N. benthamiana*, our results suggest that MoVcpo may induce multiple rice defense responses against *M. oryzae*.

### MoVcpo is highly induced during the early stages of *M. oryzae* infection

**Figure 6:**
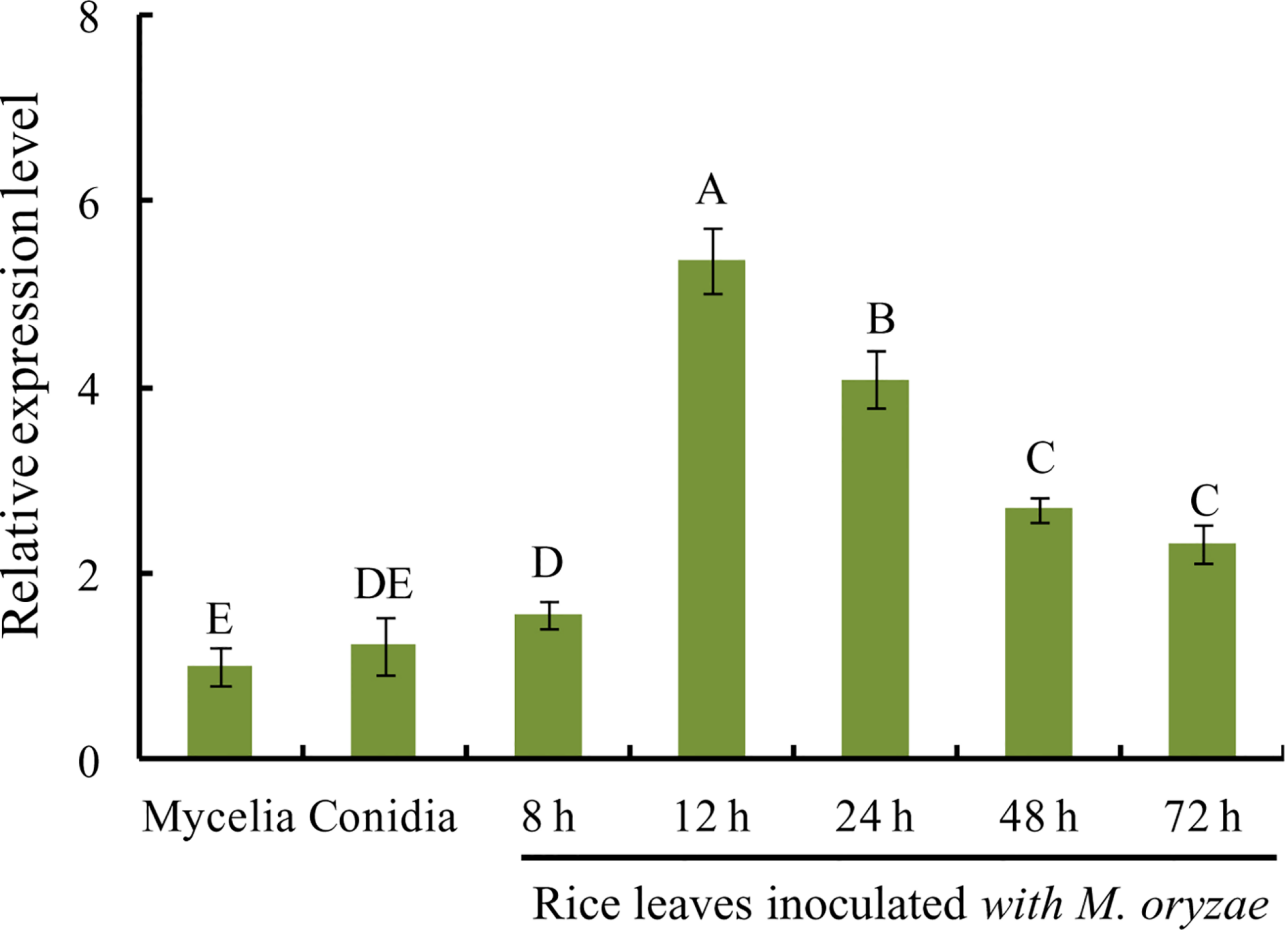
RT-qPCR analysis of *MoVcpo* expression at different developmental and infective stages. The fungal constitutive gene *Moactin* was used as internal reference. Values are the means ( ± SE) based on three independent experiments and bars indicate standard deviations. Different letters indicate statistical significance (*p* < 0.05) using Duncan’s new multiple range method.

To investigate *MoVcpo* expression pattern in *M. oryzae* at different developmental and infection stages, RT-qPCR analysis was performed on samples of vegetative mycelia, conidia, and infected leaves after inoculation with *M. oryzae* conidia. The expression level of *MoVcpo* was significantly increased in rice leaves during the early stage of *M. oryzae* infection at 12 h and 24 h (**Figure 6**). However, the expression levels of *MoVcpo* in conidia or mycelia were significantly lower than those in the infection stages. These results suggest that *MoVcpo* is highly induced at the early infection stages, indicating that MoVcpo may play an important role in *M. oryzae*-rice interaction.

### MoVcpo regulates conidiation, conidia germination, and responses to multiple stresses

**Figure 7:**
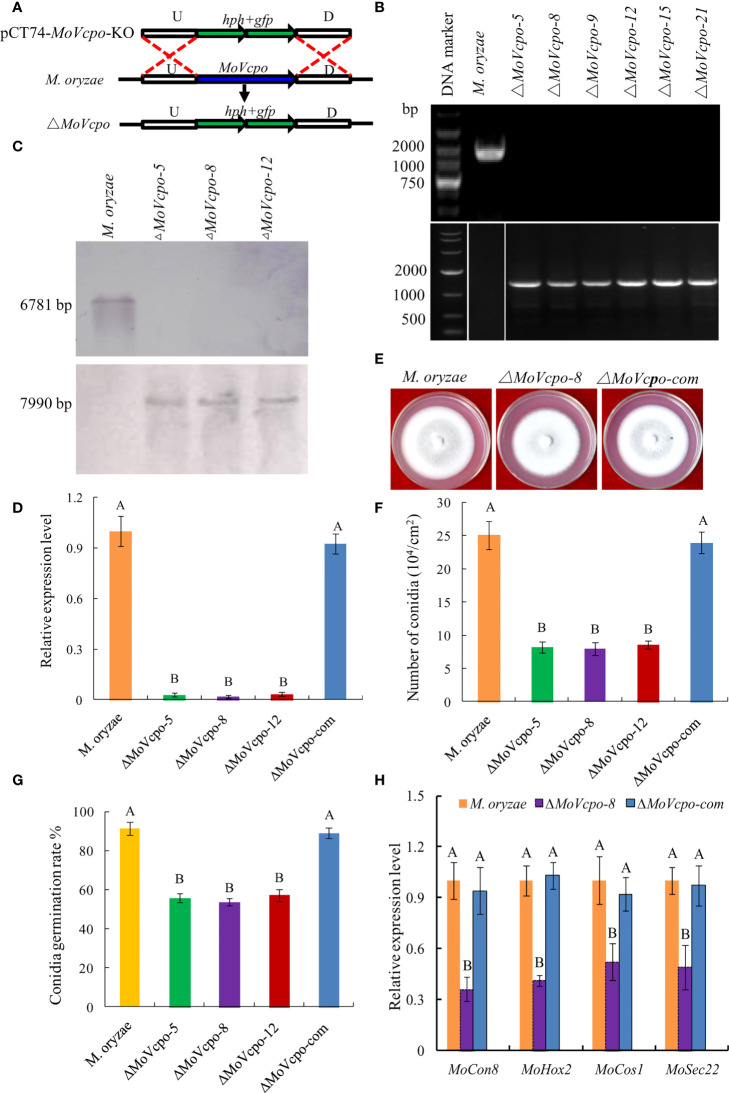
Construction, confirmation and characterization of *MoVcpo* deletion and complementation mutants. **(A)** Schematic strategy used for generation of *MoVcpo* deletion mutant according to homologous recombination. U, upstream flanking region of *MoVcpo*; D, downstream flanking region of *MoVcpo*. **(B)** Colony morphologies on YDA medium. Photos were taken at 5 d after incubation. **(C)** PCR confirmation using *MoVcpo* (Upper) and *hph* (Lower) as probes. **(D)** Southern blot validation using *MoVcpo* (Upper) and *hph* (Lower) as probes, respectively. **(E)** RT-qPCR detection of *MoVcpo* expression in the deletion mutants and complementation strain. **(F)** Conidiation. **(G)** Conidia germination rate. **(H)** RT-qPCR analysis of the conidiation-related genes. *M. oryzae*, the wide-type strain; Δ*MoVcpo*-*5*, Δ*MoVcpo-8* and Δ*MoVcpo-12*, *MoVcpo* deletion mutants; Δ*MoVcpo*-com, *MoVcpo* complementation strain. Values are the means ( ± SE) based on three independent experiments and bars indicate standard deviations. Different letters indicate statistical significance (*p* < 0.05) using Duncan’s new multiple range method.

To investigate the role of *MoVcpo* in *M. oryzae*, we deleted *MoVcpo* by replacing it with the hygromycin resistance gene *hph* through homologous recombination (**Figure 7A**). A screen of 56 hygromycin-resistant stable transformants by PCR identified six *MoVcpo* mutants (**Figure 7B**). Three mutants (Δ*MoVcpo-5*, Δ*MoVcpo-8* and Δ*MoVcpo-12*) were further confirmed to lack *MoVcpo* by Southern blot analysis using a *hph*-specific probe and a *MoVcpo*-specific probe (**Figure 7C**), and by RT-qPCR (**Figure 7D**). All three deletion mutants displayed identical phenotypes; they were highly similar to the wild type (WT) in colony growth and the morphology of mycelia and conidia, but exhibited significantly reduced conidiation and conidia germination rate on YDA medium compared to the wild type (WT) (**Figures 7E–G**, and **Supplementary Figure 7**). Therefore, we randomly selected Δ*MoVcpo-8* as the representative for further analyses. The expression of conidiation-related genes, including *MoHox2*, *MoCon1*, *MoCon8*, *MoCos1* and *MoSec22*, was significantly reduced in Δ*MoVcpo*-8 (**Figure 7H**). To further test if the effect was exclusively due to the deletion of *MoVcpo*, a complementation strain (Δ*MoVcpo*-com) was constructed based on Δ*MoVcpo*-8 and its conidiation and conidia germination rate was similar to the WT (**Figure 7** and **Supplementary Figures 7**). These results indicated that *MoVcpo* is dispensable for fungal growth, but affects spore formation and conidia germination in *M. oryzae*.

**Figure 8:**
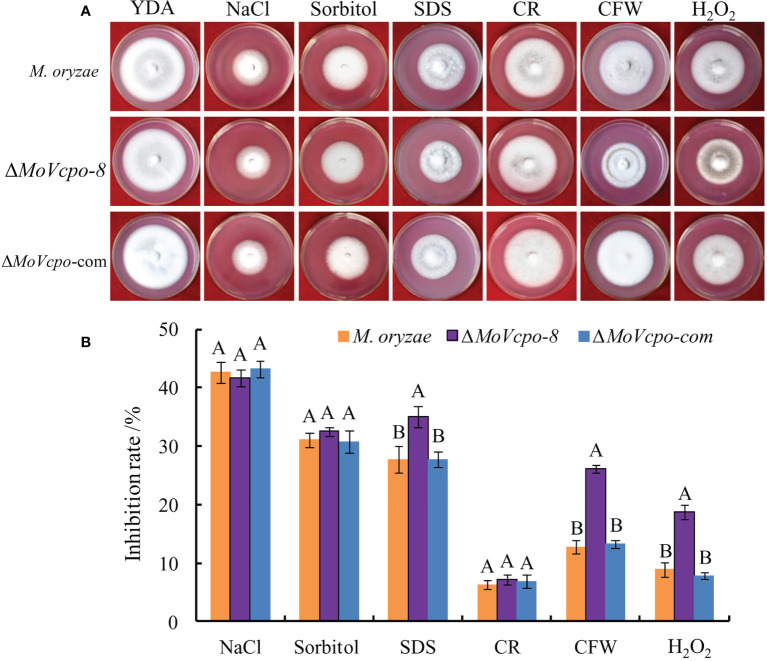
*MoVcpo* deletion affects various stress response of *M. oryzae.* **(A)** Colony morphology of the wild type, Δ*MoVcpo*-8, and Δ*MoVcpo*-com supplemented with different stressors on YDA plates. YDA, yeast-extract dextrose agar medium; CR, Congo red. CFW, calcofluor white. **(B)** The inhibition rate was calculated by comparing the colony diameter on treatments with that of YDA without treatment. The inhibition rate (%) = [(diameter of untreated colony - diameter of treated colony)/diameter of untreated colony] × 100. Data are mean ± SE from three independent replicates. Different letters indicate significant difference at *p*<0.01 level by Duncan’s new multiple range method.

To investigate whether *MoVcpo* is involved in stress responses, the WT, Δ*MoVcpo*-8, and Δ*MoVcpo*-com strains were grown on YDA supplemented with different stress conditions. As shown in **Figure 8A**, compared with the WT, Δ*MoVcpo*-8 was more sensitive to cell wall stresses imposed by CFW and SDS. The results also showed that the growth of the *MoVcpo* mutant was markedly inhibited by H_2_O_2_ (**Figure 8B**). These results indicated that the loss of *MoVcpo* affects cell wall integrity and sensitivity to osmotic stress.

### MoVcpo contributes to *M. oryzae* virulence

**Figure 9:**
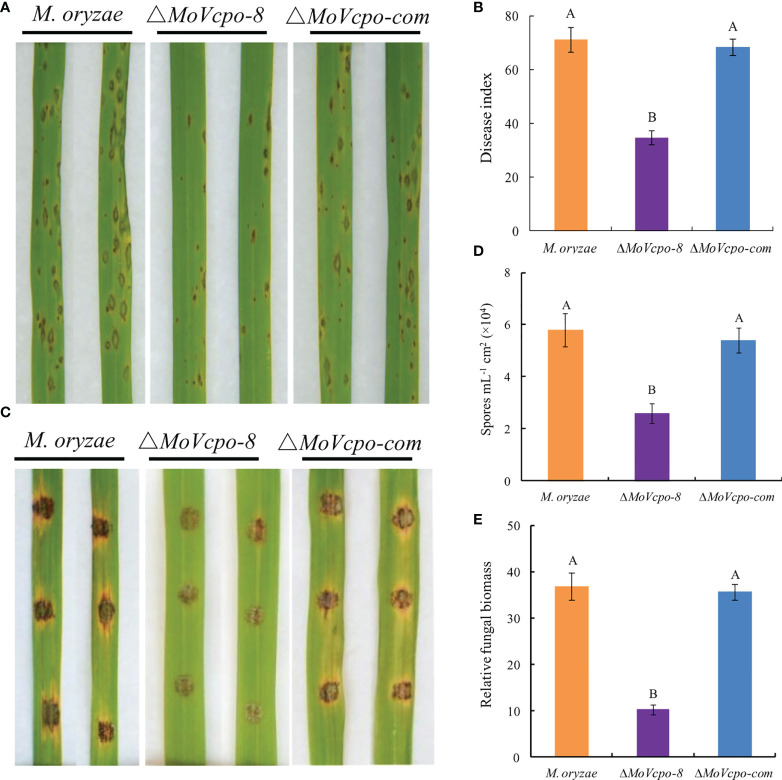
Pathogenicity assay of *MoVcpo* deletion mutant and complementation strain. **(A)** Virulence bioassay on rice cultivar cv. CO39 conducted by the spraying inoculation method. The symptoms of rice plants were investigated 7 dpi and photographed. **(B)** Disease index of rice blast in spraying inoculation bioassay. **(C)** Virulence bioassay on rice plants conducted by the punch inoculation method. Leaves were photographed 10 dpi. **(D)** Sporulation on the punch-inoculated leaves. Samples were taken for the assays 10 dpi. **(E)** Relative biomass assay on the punch-inoculated leaves. The relative fungal growth was measured by [2^[CT(^ *^OsUbiquitin^* ^)-CT(^ *^MoPot2^* ^)]^×10000] using DNA-based qPCR. Rice seedlings at the fourth-leaf stage were inoculated with the conidial suspensions (1×10^5^ conidia/mL) of the wide type, *MoVcpo* deletion mutant, and complementation strain. Values are the means from three independent experiments and bars indicate standard deviations. The letters above the bars are significantly different at 0.01 level using Duncan’s multiple range test.

To investigate whether *MoVcpo* is related to the virulence of *M. oryzae*, the susceptible rice cultivar CO39 was treated with conidia suspensions of WT, Δ*MoVcpo*-8 and Δ*MoVcpo*-com, respectively, by spray inoculation method. After 7 dpi, only a small number of disease spots formed on rice leaves inoculated by Δ*MoVcpo*-8, while more typical gray centered blast lesions and merged large lesions appeared on the leaves inoculated by the WT and Δ*MoVcpo*-com (**Figure 9A** and **Supplementary Table 3**). Consistent with the lesion results, the disease index was 41.2% lower on Δ*MoVcpo*-8-inoculated rice leaves in comparison with WT- and Δ*MoVcpo*-com- inoculated ones (**Figure 9B**). To further determine the impact of *MoVcpo* deletion on the virulence, we then assessed fungal growth in rice using the punch inoculation method, which is more suitable for measuring the basal resistance levels of rice plants than the spray inoculation method (Ono et al., 2001). The assays showed that Δ*MoVcpo*-8 caused smaller disease lesions on rice leaves than the WT and Δ*MoVcpo*-com (**Figure 9C**). Consistent with the lesion size result, fewer spores were produced on the Δ*MoVcpo*-8-inoculated rice leaves than those on the WT- and Δ*MoVcpo*-com- inoculated ones (**Figures 9D**). Furthermore, the relative fungal biomass was lower in Δ*MoVcpo*-8-infected leaves as measured by DNA-based qPCR (**Figure 9E**). These results showed that *MoVcpo* deletion leads to reduced virulence of *M. oryzae* to rice plants, indicating that *MoVcpo* plays an important role in virulence.

### MoVcpo deletion activates plant immunity responses in rice

To investigate whether the attenuated virulence of *MoVcpo* deletion mutant was related to ROS accumulation in rice, we conducted quantitative analyses of the production of H_2_O_2_. As shown in **Supplementary Figure 8A**, H_2_O_2_ accumulation was significantly higher in rice leaves inoculated with Δ*MoVcpo-8* than in those inoculated with the WT and Δ*MoVcpo*-com. We further determined the expression of four well-known defense-related genes, *OsEDS1*, *OsAOS2*, *OsWRKY45*, and *OsPR1a*. The expression of *OsEDS1*, *OsWRKY45*, and *OsPR1a*, was significantly up-regulated in rice plants at 24 h after inoculation with Δ*MoVcpo-8* in comparison with the WT inoculation (**Supplementary Figure 8B**). These results showed that *MoVcpo* deletion results in enhanced defense response, further indicating that MoVcpo may act as a critical virulence factor to facilitate *M. oryzae* infection by suppressing host immunity.

## Discussion

Elicitors play an important role in induced plant resistance to fungal pathogens (Giovannoni et al., 2021; Zhang et al., 2021). Many of them are constituents of the pathogen or secreted by it, or they are released from pathogen cell walls by hydrolytic enzymes from the pathogen or the plant (Garcia-Brugger et al., 2006). Various types of elicitors have been isolated and purified from *M. oryzae*, including proteins (Kanoh et al., 1993; Schaffrath et al., 1995; Chen et al., 2012; Chen et al., 2014), cerebrosides (Koga et al., 1998), chitin (Ren and West, 1992), and N-acetylchitooligosaccarides (Yamada et al., 1993; Shinya et al., 2022), all of which seem to activate defense-related gene expression, and induce the defense response against *M. oryzae* infection in rice. In this study, we isolated and purified a novel elicitor, MoVcpo, from *M. oryzae.* Similar to previously reported elicitors, MoVcpo triggers strong defense response in both tobacco and rice plants, including the accumulation of ROS, and up-regulated expression of defense-related genes. Moreover, we found that MoVcpo also functioned as a virulence factor to suppress host immunity and therefore was required for the full virulence of *M. oryzae*.

It is well documented that ROS generation is an important characteristic of plant-induced immunity in response to elicitor stimulation (Nanda et al., 2010). In this study, the results from the DAB staining and quantitative analyses showed that the elicitor MoVcpo resulted in a significant increase in H_2_O_2_ and O_2_^.-^ content, which further induced an HR in *N. benthamiana* (**Figure 2**). An increase in H_2_O_2_ and O_2_^.-^ content after MoVcpo treatment was also observed in rice leaves (**Figure 4**). Similar results were also observed in previous studies of elicitors. For example, PeBL2, an elicitor-bioactivity protein isolated from *Brevibacillus laterosporus*, triggered an early defense response in *N. benthamiana* as revealed by ROS accumulation (Jatoi et al., 2019). Consistent with these findings, the activity of LOX, which is an important ROS-generation source, increased markedly after MoVcpo treatment, which is one of important ROS-generation source. These results indicated that MoVcpo induced ROS accumulation by coordinating oxidative burst through increasing the activity of ROS-generation enzyme.

Plants possess a complex defense system that includes both broad and specific responses such as protein phosphorylation, ion fluxes, activation of MAPK cascades, activation of WRKY transcription factors, and transcription of defense-related genes (Shen et al., 2017). Our results showed that genes encoding transcription factor (OsWRKY45), PR protein (OsPR1a), and OsMAPK6 for MAPK signaling pathway, were all up-regulated significantly at early stage after treatment with MoVcpo. Interestingly, the marker genes for both SA-signaling pathway (*OsEDS1*) and JA-signaling pathway (*OsAOS2*, *OsPBZ1*) were significantly up-regulated simultaneously after elicitation, suggesting that both JA- and SA-dependent responses are activated by MoVcpo. Consistent with these results, MoVcpo also activated the SA- and JA-mediated defense pathways in *N. benthamiana*. Similarly, induced expression of the marker genes involved in JA/SA signaling was reported in *Vitis rupestris* following the treatments with both elicitor flg22 and harpin (Chang and Nick, 2012). PeFOC1, an elicitor isolated from *Fusarium oxysporum*, also activates SA and JA signaling pathways, and triggers the immune response and systemic acquired resistance in tobacco (Li et al., 2019). Taken together, MoVcpo induced multiple signaling pathways in rice against fungal infection. However, further investigations are needed to understand the role of JA/SA signaling in rice defense response triggered by the elicitor MoVcpo.

Recent studies show that many elicitors exhibit an induced expression pattern in pathogen-plant interaction (Chen et al., 2013; Wang et al., 2016; Pradhan et al., 2021). Usually, the gene expression significantly increases during fungal infection, such as MoHrip1 and Rbf1 in *M. oryzae* (Nishimura et al., 2016; Nie et al., 2019), SsSSVP1 in *Sclerotinia sclerotiorum* (Lyu et al., 2016), and VdPEL1 in *Verticillium dahliae* (Yang et al., 2018). The transcript levels of MoVcpo also showed a significant increase at the early stage of *M. oryzae* infection, suggesting that it may play an important role in the fungal infection process. To further characterize the function of *MoVcpo*, we deleted *MoVcpo* gene and found that the *MoVcpo* deletion resulted in compromised fungal cell wall integrity and increased sensitivity to osmotic stress, and, importantly, reduced pathogenicity of *M. oryzae* to rice plants (**Figure 9**). The expression levels of defense-related genes were obviously higher in Δ*MoVcpo* -inoculated rice leaves than those in the WT- and Δ*MoVcpo*-com-inoculated rice leaves (**Supplementary Figure 7**). Similarly, MoHrip2 and MSP1, two other *M. oryzae* elicitors, can also activate rice defense responses during *M. oryzae* inoculation, and their deletion mutants exhibit significantly decreased virulence, suggesting that they also function as both virulence factors and elicitors in *M. oryzae* (Jeong et al., 2007; Chen et al., 2014; Wang et al., 2016; Wang et al., 2017; Nie et al., 2019). Together with the above studies, our results provide new evidences that elicitors can function as both PAMPs to activate plant immunity and a virulence factor to promote fungal infection.

Vanadium chloroperoxidases (Vcpo) are widely present in prokaryotes and a group of common terrestrial fungi (the dematiaceous Hyphomycetes) and are involved in the specific synthesis of chlorinated antibiotics. Vcpo proteins possess an oxidometalate (vanadate) as a prosthetic group, making them very resistant to oxidative inactivation (Bernhardt et al., 2011; Dong et al., 2017; McKinnie et al., 2018). Barnett et al. (1997) found that Vcpo is excreted by the growing hyphae of *Curvularia inequalis*. Further investigations suggested that Vcpo can oxidize the waxy protective cuticle layer on the leaves of plant and/or degrade the cell walls of the plants to facilitate the penetration of the fungus through the leaf cuticle/and or plant cell to reach nutrients in the cell (Wever and Barnett, 2017). In many pathogenic fungi, Vcpo is also considered to play an important role in the formation of CHCl_3_ (Wever and Barnett, 2017). Although some studies in recent years have increased our understanding of halogenase biochemistry, the function of Vcpo is often disputed and still remains poorly understood. In this study, we revealed the function of Vcpo in *M. oryzae* as an elicitor, which provides useful clues for further studies to dissect Vcpo function in pathogenic fungi.

Taken together, in this study, we found the vanadium chloroperoxidase Vcpo in *M. oryzae* can function as both an elicitor to activate plant immunity and a virulence factor to promote fungal infection, which adds to our understanding of the fungi-plant interaction. Further studies are required to identify the host receptor for MoVcpo in rice leaves to elucidate the mechanism of MoVcpo-induced/suppressed resistance in rice.

## Data availability statement

The datasets presented in this study can be found in online repositories. The names of the repository/repositories and accession number(s) can be found in the article/**Supplementary Material**.

## Author contributions

YN, XfZ, HL, X-LC, and YL conceived, wrote, reviewed, edited the manuscript, and designed the experiments. YN, GL, JL, XsZ, YZ, QS, HL, and YL performed the experiments and analyzed the data. YN, GL, JL, QS, and YL contributed reagents, materials, and analysis tools. All authors have read and agreed to the published version of the manuscript.

## Funding

This research was funded by Natural Science Foundation of Guangdong Province (2021A1515010643), Guangzhou Science and Technology Program (202206010027), the National Natural Science Foundation of China (31671968), Project for Key Technology R&D Innovation Team in Modern Agriculture, Guangdong Province (2021KJ134), and China Agriculture Research System of MOF and MARA (CARS-31).

## Conflict of interest

The authors declare that the research was conducted in the absence of any commercial or financial relationships that could be construed as a potential conflict of interest.

## Publisher’s note

All claims expressed in this article are solely those of the authors and do not necessarily represent those of their affiliated organizations, or those of the publisher, the editors and the reviewers. Any product that may be evaluated in this article, or claim that may be made by its manufacturer, is not guaranteed or endorsed by the publisher.
