# Supplementary material for: A novel elicitor MoVcpo is necessary for the virulence of Magnaporthe oryzae and triggers rice defense responses
Source: Front Plant Sci. 2022 Oct 17;13:1018616. doi: 10.3389/fpls.2022.1018616 (PMC9619064; doi:10.3389/fpls.2022.1018616)
Supplement: Supplementary file 1 [file DataSheet_1.pdf]

Supplementary Material

1 Supplementary Figures and Tables

1.1 Supplementary Figures

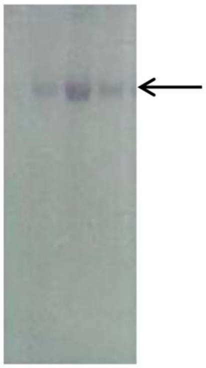

Supplementary Figure 1. SDS-PAGE analysis of the purified elicitor protein using PAS staining. Fifteen μg total proteins per lane was loaded. The elicitor band was indicated by an arrow.

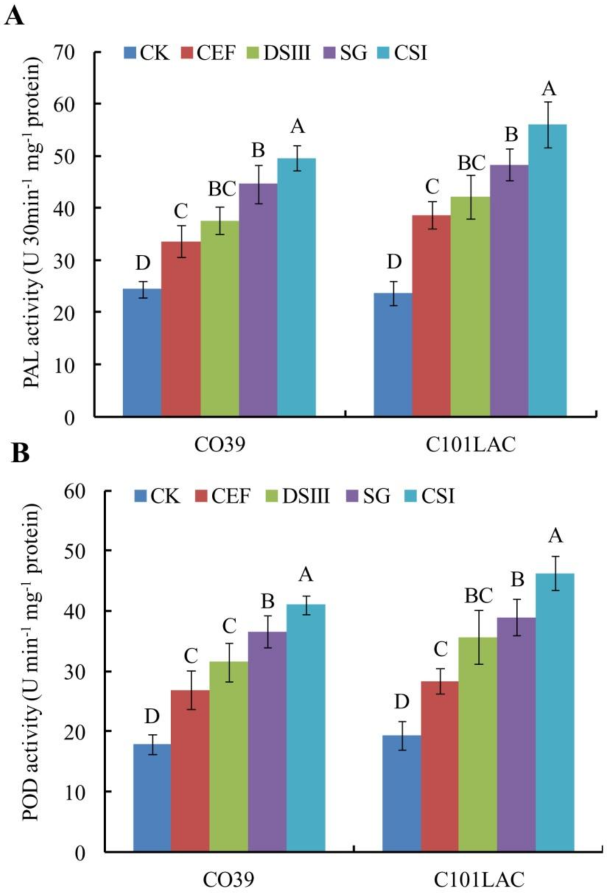

Supplementary Figure 2. The activities of PAL (A) and POD (B) in rice leaves treated by elicitor fractions from *M. oryzae*. Five μL of different elicitor fractions (50 μg/mL) was mounted onto a punch-inoculated spot of 2 mm diameter on the unexcised 4th rice leaves. CK, TH buffer; CEF, crude elicitor fraction; DSIII and SG, the elicitor fractions purified by DEAE-Sepharose FF and Sephadex G-100 chromatography; CSI, the purified elicitor. Values are the means (±SE) based on three independent experiments and bars indicate standard deviations. Different letters indicate statistical significance ($p < 0.05$) using Duncan's new multiple range method.

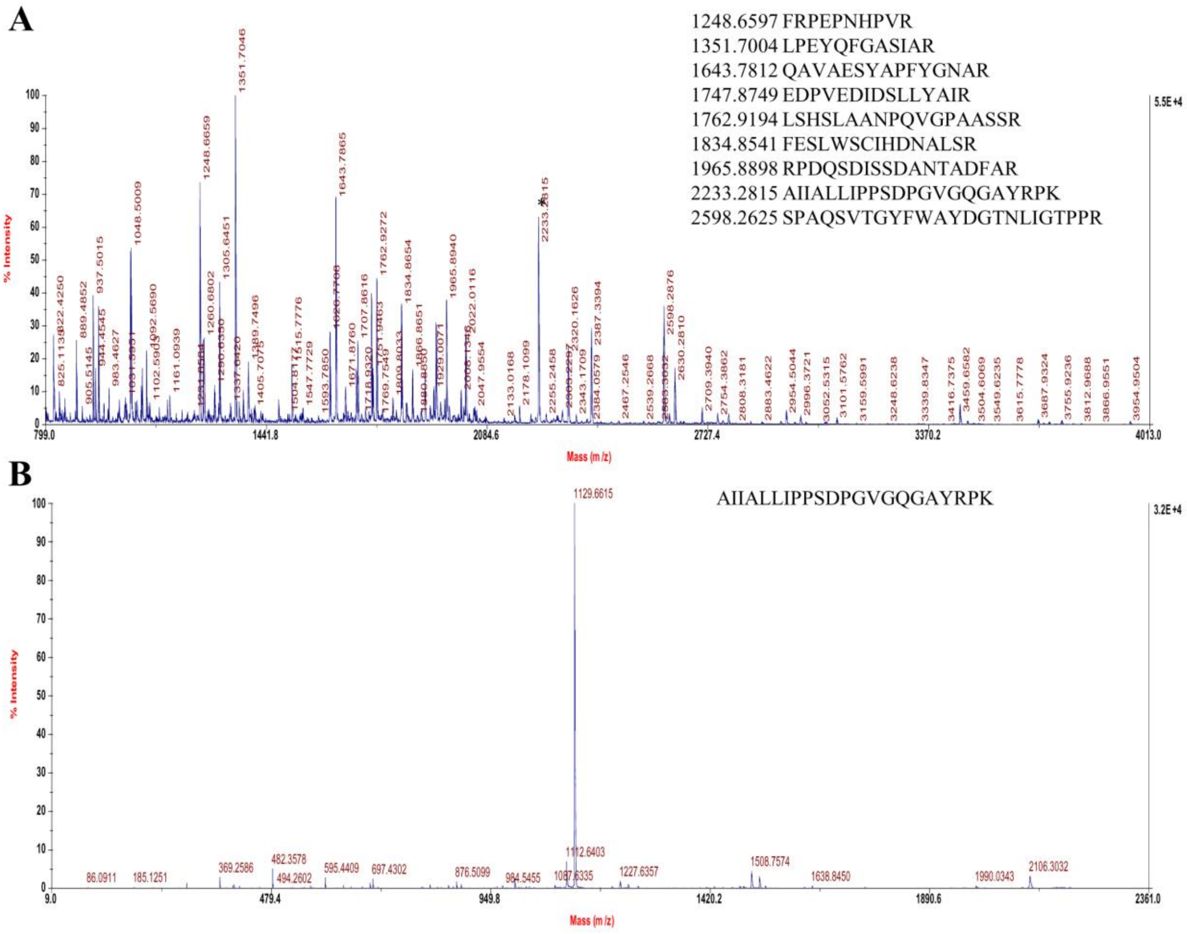

Supplementary Figure 3. Identification of the purified elicitor by MS. (A) The MS spectra. The matched peptides and their corresponding peaks are listed in the map. The ion 2233.28 marked with an asterisk was analyzed by MS/MS. (B) MS/MS spectra of ion 2233.28. The protein excised from CBB-staining gel was digested with trypsin, and the resulting peptides were analyzed using the 4700 Proteomic Analyzer. The corresponding peptide sequence is shown. The protein was identified as vanadium chloroperoxidase after database searching.

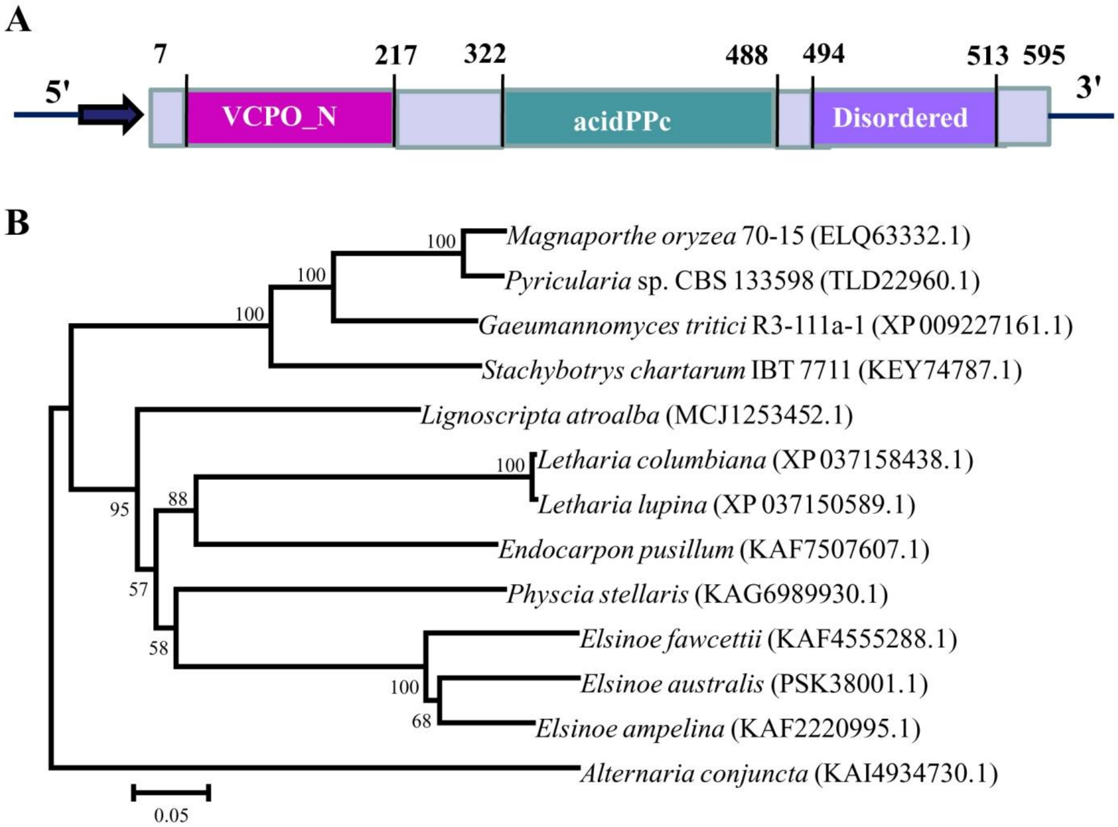

Supplementary Figure 4. Bioinformatics analysis of MoVcpo. (A) Schematic structure of the MoVcpo protein. The domains were predicted by Pfam. (B) Phylogenetic analysis of the MoVcpo protein. Maximum likelihood tree was built from MoVcpo and its orthologous proteins from twelve fungal pathogens.

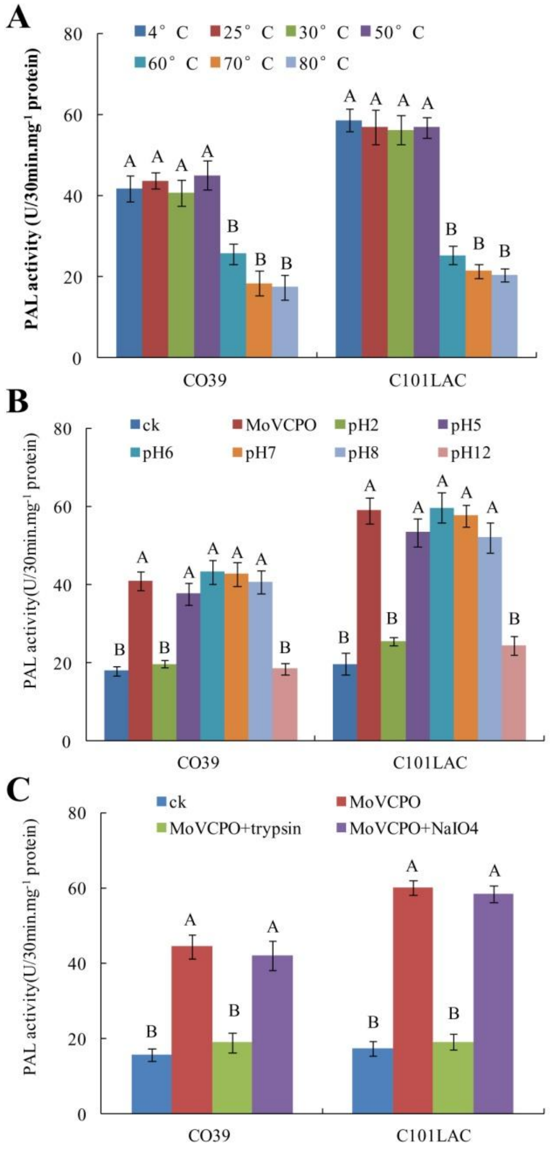

Supplementary Figure 5. Induction of PAL activities in rice leaves after application of heat- (A), chemical- (B), and enzyme- (C) treated MoVcpo. The elicitor concentration of each treatment is 50 μg/mL. Values are the means (±SE) based on three independent experiments and bars indicate standard deviations. Different letters indicate statistical significance ($p < 0.05$) using Duncan's new multiple range method. For treatments see materials and methods.

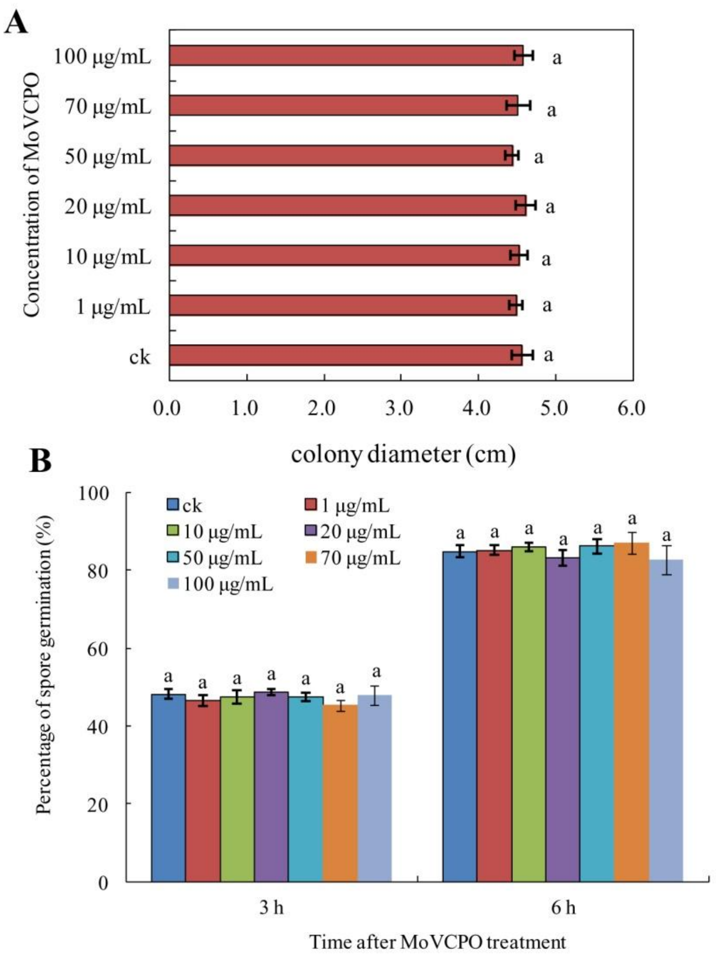

Supplementary Figure 6. Effect of MoVcpo on mycelial growth (A) and spore germination (B) of *M. oryzae*. Values are the means (±SE) based on three independent experiments and bars indicate standard deviations. Different letters indicate statistical significance ($p < 0.05$) using Duncan's new multiple range method.

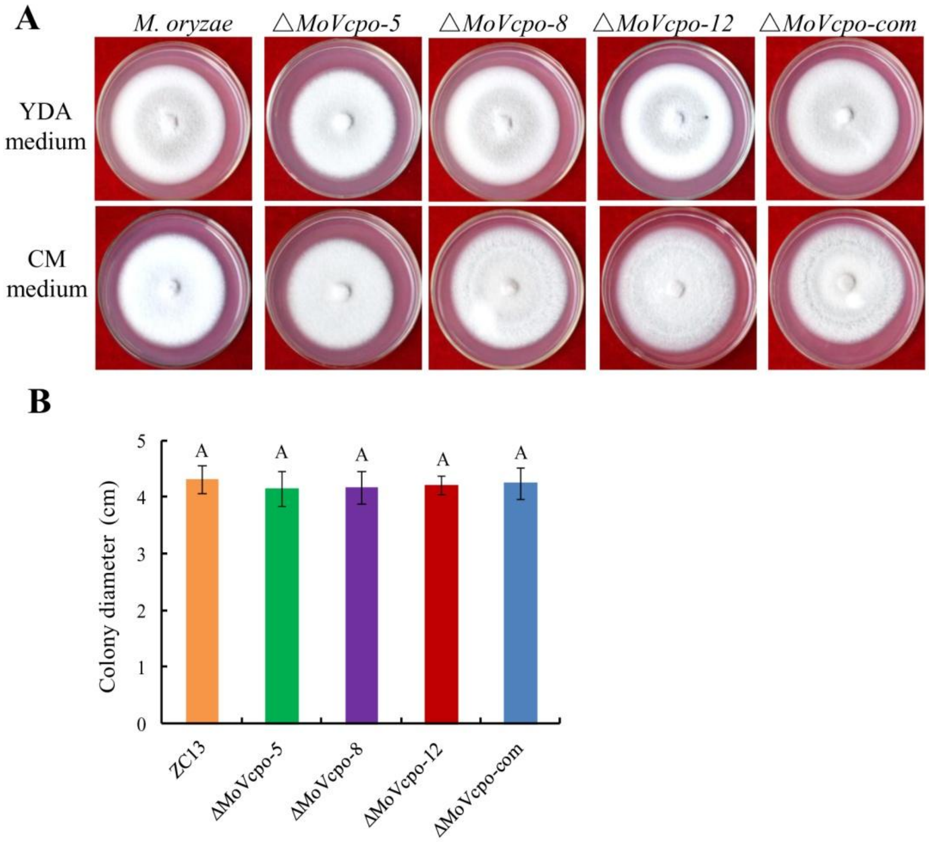

Supplementary Figure 7. Phenotypes in colony of *MoVcpo* deletion mutants and complementation strain. (A) Colony morphology. (B) Colony diameter on YDA medium. *M. oryzae,* the wide-type strain; Δ*MoVcpo-5,* Δ*MoVcpo-8* and Δ*MoVcpo-12*, *MoVcpo* deletion mutants; Δ*MoVcpo*-com, *MoVcpo* complementation strain. Images were taken at 5 dpi. Values are the means (±SE) based on three independent experiments and bars indicate standard deviations. Different letters indicate statistical significance ($p < 0.05$) using Duncan's new multiple range method.

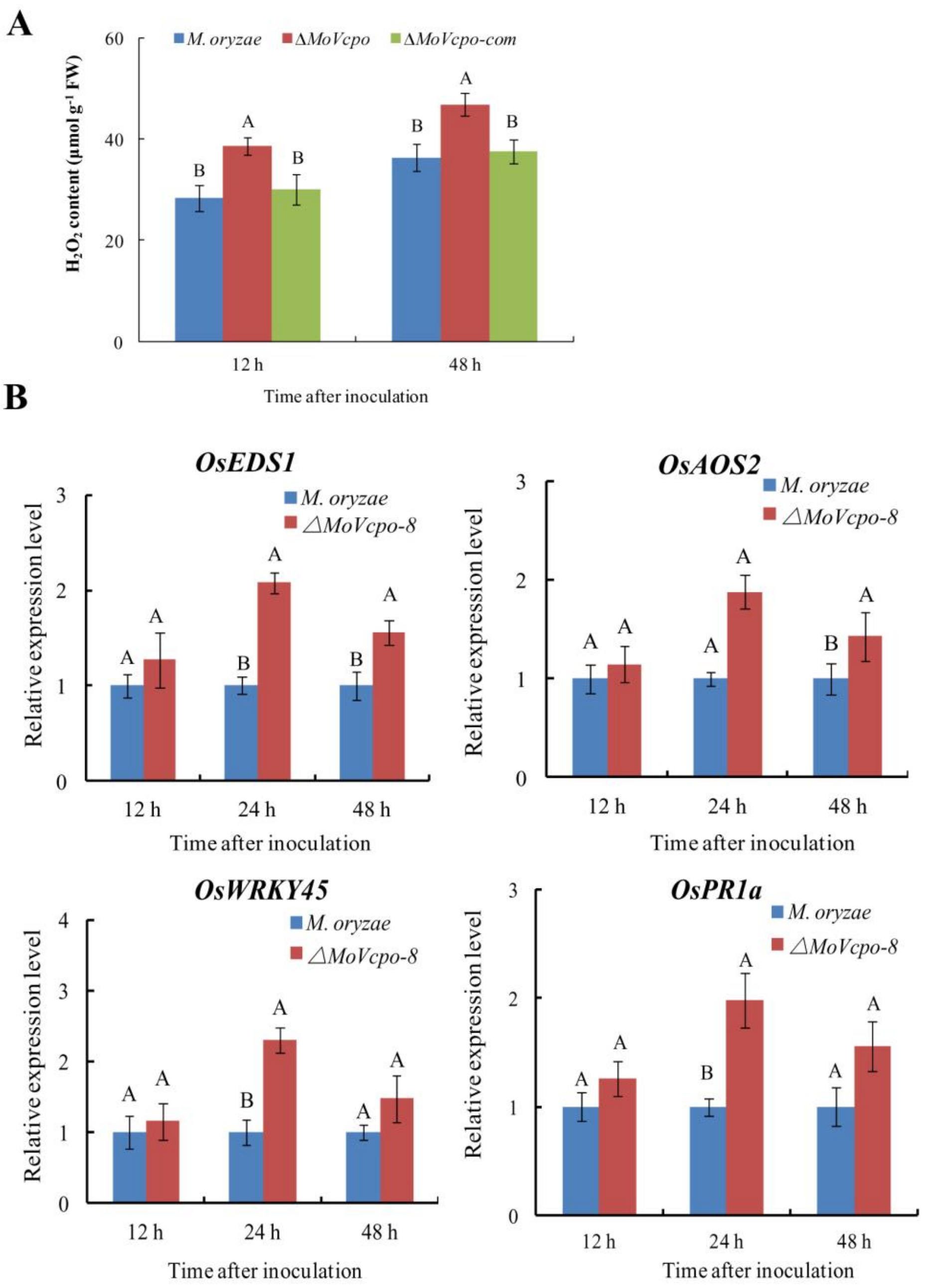

Supplementary Figure 8. H_2O_2 production and transcription patterns of four defense-related genes in rice cultivar cv. CO39 after inoculated with the wide type and Δ*MoVcpo-8* as determined by RT-qPCR. The rice constitutive gene *Osactin* was used as internal reference. Values are the means (±SE) based on three independent experiments and bars indicate standard deviations. Different letters indicate statistical significance (p < 0.05) using Duncan's new multiple range method.

1.2 Supplementary Tables

Supplementary Table 1. Primers used in this study

**Primer name**	**Primer Sequence 5'-3'**	**Reference**
Construction and confirmation of *MoVcpo* deletion and complementation mutants		
MoVcpo-upF	GGTACCGTCCAAAGCAAGATGCCCCTGTGTT	Present work
MoVcpo-upR	GGGCCCTGTGACACAAGCTTGAAGTTCAATG	Present work
MoVcpo-downF	GAATTCGAAGCAAAGGTGTCAGTGGTTTAAC	Present work
MoVcpo-downR	TCTAGACATGCTAAAGAATACGCCAAGAGTC	Present work
hph-F	TTCTGCGGGCGATTTGTGTA	Present work
hph-R	AAAAAGCCTGAACTCACCGC	Present work
MoVcpo-F	ATCCCCATTCTTCCTCCCAC	Present work
MoVcpo-R	AACAAGCCCTCCCTGTCGAA	Present work
hph-porobe -F	TGCTGCTCCATACAAGCCAA	Present work
hph-porobe -R	GACATTGGGGAGTTCAGCGA	Present work
MoVcpo-porobe-F	AGCCTGCCGAGTACAACAAC	Present work
MoVcpo-porobe-R	AGGAATGAGAAGCGCGATGA	Present work
MoVcpo-comF	GGAATTCGCCGCCTCATTGTTGTCTGT	Present work
MoVcpo-comR	GCTCTAGAACAGTTGTAAGGTGGTTGGCT	Present work
DNA-based qPCR analysis of fungal biomass in punch inoculation		
q*MoPot2*-F	ACGACCCGTCTTTACTTATTTGG	Parker *et al.*, 2012
q*MoPot2*-R	AAGTAGCGTTGGTTTTGTTGGAT	Parker *et al.*, 2012
q*OsUbiquitin*-F	TTCTGGTCCTTCCACTTTCAG	Parker *et al.*, 2012
q*OsUbiquitin*-R	ACGATTGATTTAACCAGTCCATGA	Parker *et al.*, 2012
RT-qPCR analysis for *MoVcpo* in *M. oryzae*		
q*MoActin*-F	TCGACGTCCGAAAGGATCTGT	Pan *et al.*, 2019
q*MoActin*-R	ACTCCTGCTTCGAGATCCACATC	Pan *et al.*, 2019
q*MoVcpo*-F	TCCAGACTGACAACCACCTCCT	Present work
q*MoVcpo*-R	AAAGTATCCCGTGACCGACTGC	Present work
RT-qPCR analysis of defense related genes in rice		
q*OsPR1a*-F	TCTTCATCACCTGCAACTACTC	Pan *et al.*, 2019
q*OsPR1a*-R	ATTCATCGGATTTATTCTCACC	Pan *et al.*, 2019
q*OsPBZ1*-F	CTACTATGGCATGCTCAAGAT	Pan *et al.*, 2019
q*OsPBZ1*-R	ATAGAAAGGCACATAAACACAA	Pan *et al.*, 2019
q*OsAOS2*-F	GCGAGAGACGGAGAACCC	Nguyễn *et al.*, 2014
q*OsAOS2*-R	CGACGAGCAACAGCCTTC	Nguyễn *et al.*, 2014
q*OsEDS1*-F	CAGGAGAGGCAGTGTTAATCAG	Nguyễn *et al.*, 2014
q*OsEDS1*-R	GCAAGCGGAGTAAGTGGTATG	Nguyễn *et al.*, 2014
q*OsMAPK6*-F	GATACATTCGCCAACTTCC	Nguyễn *et al.*, 2014
q*OsMAPK6*-R	CAGTGATGCCAGGTAAGG	Nguyễn *et al.*, 2014
q*OsWRKY45*-F	CCGAAGAATCATGGATGGAC	Uji *et al.*, 2019
q*OsWRKY45*-R	GCGCTGCCGCTAATTATTTC	Uji *et al.*, 2019

q*OsActin*-F	GAGTATGATGAGTCGGGTCCAG	Uji *et al.*, 2019
q*OsActin*-R	ACACCAACAATCCCAAACAGAG	Uji *et al.*, 2019

Reference

Nguyễn, P. V., Bellafiore, S., Petitot, A. S., Haidar, R, Bak, A., Abed, A., et al. (2014). Meloidogyne incognita-rice (*Oryza sativa*) interaction: a new model system to study plant-root-knot nematode interactions in monocotyledons. *Rice (N Y)* 7(1):23. doi: 10.1186/s12284-014-0023-4.

Pan, Y., Pan, R., Tan, L., Zhang, Z., and Guo, M. (2019). Pleiotropic roles of O-mannosyltransferase MoPmt4 in development and pathogenicity of *Magnaporthe oryzae*. *Curr. Genet.* 65(1):223-239. doi: 10.1007/s00294-018-0864-2.

Park, C. H., Chen, S., Shirsekar, G., Zhou, B., Khang, C. H., Songkumarn, P., et al. (2012). The *Magnaporthe oryzae* effector AvrPiz-t targets the RING E3 ubiquitin ligase APIP6 to suppress pathogen-associated molecular pattern-triggered immunity in rice. *Plant Cell*. 24(11):4748-62. doi: 10.1105/tpc.112.105429

Uji, Y., Kashihara, K., Kiyama, H., Mochizuki, S., Akimitsu, K., and Gomi, K. (2019). Jasmonic acid-induced VQ-motif-containing protein OsVQ13 influences the OsWRKY45 signaling pathway and grain size by associating with OsMPK6 in rice. *Int J Mol Sci.* 20(12):2917. doi: 10.3390/ijms20122917.

Supplementary Table 2. Induction of rice resistance against blast disease with different concentrations of the elicitor *MoVcpo*[a]

Concentration (μg/mL)	Disease index (DI)		Reduction of DI (%)[b]	
	CO39	C101LAC	CO39	C101LAC
CK	75.7±3.1a	28.5±2.7a	-	-
1	67.5±2.6a	25.1±1.4a	10.8±5.2c	11.9±2.8c
10	51.3±2.1b	20.4±2.3b	32.2±3.8b	28.4±2.7b
20	47.6±1.9b	19.4±2.0b	37.1±2.6b	31.9±2.3b
50	38.6±3.7c	16.5±1.6c	49.0±3.1a	42.1±2.6 a
70	34.7±4.1cd	15.4±2.4c	54.2±3.8a	46.0±3.6a
100	32.8±2.8d	14.8±2.1c	56.6±4.2a	48.1±3.4a

a) Rice seedlings at the fully developed fourth-leaf stage were sprayed with the elicitor solution at the time zero, and challenged with fresh *M. oryzae* spores (1×10^5 conidia/mL, containing 0.02% v/v Tween 20) at the 2nd day. Control plants were sprayed with TH buffer and inoculated in the same way as the elicitor-treated plants. The data were means (± SE) from three independently biological experiments with 30 seedlings. The data in the same column followed by different letters are significantly different at 0.05 level.

b) Reduction of disease index indicates reduced blast disease severity.

Supplementary Table 3. Response of rice cultivar CO39 plants to different *M. oryzae* strains treatments

Treatment	Disease index
M. oryzae	75.7 ±3.1a
△*MoVcpo-8*	44.5 ±2.6b
△*MoVcpo-com*	77.1 ±2.1a

Rice seedlings at the fully developed fourth-leaf stage were sprayed with fresh spores (1×10^5 conidia/mL, containing 0.02% v/v Tween 20). The data were means (± SE) from three independently biological experiments with 30 seedlings. The data in the same column followed by different letters are significantly different at 0.05 level.
